# Public Reporting in Cardiothoracic Surgery: Does Transparency Improve Care?

**DOI:** 10.3390/medsci14040461

**Published:** 2026-08-06

**Authors:** Vasileios Leivaditis, Francesk Mulita, Sofoklis Mitsos, Periklis Tomos, Nikolaos Kontodimopoulos, Elias Liolis, Konstantinos Nikolakopoulos, Irida Pano, Theodora Skoura, Efstratios Koletsis, Nikolaos G. Baikoussis, Anastasios Sepetis

**Affiliations:** 1 Department of Cardiothoracic and Vascular Surgery, Westpfalz Klinikum, 67655 Kaiserslautern, Germany; 2 Second Department of Surgery, Medical School, Democritus University of Thrace, 68100 Alexandroupolis, Greece; 3 Department of Thoracic Surgery, Attikon General Hospital, National and Kapodistrian University of Athens, 12461 Athens, Greece; sophocmit@yahoo.gr (S.M.); periklistomos@hotmail.com (P.T.); 4 Department of Economics and Sustainable Development, Harokopio University, 17676 Athens, Greece; nkontodi@hua.gr; 5 Department of Oncology, General University Hospital of Patras, 26504 Patras, Greece; lioliselias@yahoo.gr; 6 Department of Vascular Surgery, General University Hospital of Patras, 26504 Patras, Greece; konstantinosn@yahoo.com; 7 Department of Clinical Subject, University of Medicine Tirana, 1005 Tirana, Albania; iridapano@hotmail.com; 8 Medical School, National and Kapodistrian University of Athens (NKUA), Aretaeion Hospital, 11528 Athens, Greece; tskoura@yahoo.com; 9 Department of Cardiothoracic Surgery, General University Hospital of Patras, 26504 Patras, Greece; ekoletsis@hotmail.com; 10 Department of Cardiac Surgery, Ippokrateio General Hospital of Athens, 11527 Athens, Greece; nikolaos.baikoussis@gmail.com; 11 Postgraduate Health and Social Care Management Program, Department of Business Administration, University of West Attica, 12243 Athens, Greece; tsepet@uniwa.gr

**Keywords:** transparency, public reporting, cardiothoracic surgery, ethics, outcomes, risk adjustment, quality improvement

## Abstract

**Introduction**: Transparency and public reporting have become central components of quality governance in cardiothoracic surgery, promoting professional accountability, supporting informed patient decision-making, and strengthening public trust. Their importance is particularly evident in a specialty characterized by technically demanding procedures and high clinical risk. **Aims and Objectives**: This narrative review examines the impact of public reporting of cardiothoracic surgical outcomes on clinical practice, ethical decision-making, surgeon well-being, and patient choice. It explores both the intended benefits of transparency and its potential unintended consequences. Unlike previous reviews focusing primarily on quality metrics or reporting systems, this review integrates clinical evidence with ethical analysis to propose a balanced framework for responsible transparency in cardiothoracic surgery. **Materials and Methods**: A narrative literature review was conducted using the PubMed and ScienceDirect databases. Relevant articles published between 2000 and 2026 were identified using the search terms “cardiothoracic surgery,” “public reporting,” “transparency,” and “risk adjustment.” **Results**: Public reporting has been associated with improved benchmarking, enhanced quality improvement initiatives, greater institutional accountability, and more informed patient choice. Evidence also suggests modest improvements in selected clinical outcomes. However, these benefits may be accompanied by unintended effects, including risk-averse decision-making, avoidance of high-risk cases, distortion of clinical judgment, reduced willingness to adopt innovative techniques, and potential inequities for institutions caring for more complex patient populations. **Conclusions**: Public reporting should evolve beyond the simple disclosure of performance metrics toward an ethically grounded model of transparency that is context-sensitive, appropriately risk-adjusted, and patient-centered. Such an approach should promote shared accountability, emphasize continuous quality improvement rather than punitive performance assessment, and incorporate expert clinical interpretation to ensure fairness, preserve innovation, and support equitable patient care.

## 1. Introduction

### 1.1. Why Transparency Matters in Cardiothoracic Surgery

Cardiothoracic surgery is among the highest-risk specialties in modern medicine. Most procedures are technically complex, irreversible, and associated with a substantial risk of mortality and serious morbidity [1]. Consequently, patients undergoing cardiac or thoracic surgery must make decisions under conditions of considerable uncertainty, placing exceptional trust in the expertise and judgement of their surgical team.

Whether undergoing coronary artery bypass grafting, valve surgery, or complex aortic reconstruction, patients entrust their lives to clinicians whose technical competence, clinical judgement, and experience they are often unable to assess independently [2]. This inherent information asymmetry has traditionally been addressed through professional self-regulation and the trust placed in the medical profession. Increasingly, however, transparency has emerged as a means of reducing this imbalance by providing patients with meaningful information about the qualifications, experience, and performance of the professionals responsible for their care [3].

Respect for patient autonomy further reinforces the ethical importance of transparency. Meaningful treatment decisions require patients to receive sufficient information not only about the proposed intervention but also about the context in which that care is delivered [4]. This consideration is particularly relevant in cardiothoracic surgery, where patients and clinicians frequently face difficult decisions involving complex trade-offs between survival, quality of life, procedural risk, and long-term outcomes. Individual patients may reasonably assign different values to these competing priorities, making informed decision-making an essential component of ethical surgical practice [5].

Historically, public confidence in the medical profession has been founded on the fiduciary relationship between physicians and patients, whereby clinicians are expected to act primarily in the best interests of those under their care [6]. Nevertheless, several high-profile failures in cardiothoracic surgery have exposed the limitations of professional self-regulation and intensified calls for greater external accountability and public oversight [7].

The Bristol Royal Infirmary inquiry in the United Kingdom, which followed years of unaddressed concerns regarding excess mortality in paediatric cardiac surgery, remains one of the most influential examples of the consequences of inadequate transparency and oversight [8]. Such events challenged the traditional social contract between medicine and society and highlighted the need for more robust systems of outcome monitoring, performance evaluation, and public communication to strengthen accountability while maintaining public trust.

### 1.2. Rise of Public Reporting in Surgery

The transition from professional self-regulation to public accountability represents one of the most significant developments in the governance of cardiothoracic surgery over the past several decades [1]. This transformation has been driven by the convergence of three major forces: the quality improvement movement, which emphasizes systematic measurement and continuous performance improvement; the growing recognition of patients’ rights and shared decision-making; and healthcare policy reforms aimed at improving outcomes while promoting efficiency and accountability [9].

The modern concept of quality measurement originated in industrial process improvement, where systematic evaluation of performance was used to identify variation, reduce errors, and optimize complex systems. These principles were subsequently adopted in healthcare, where the routine collection and analysis of clinical outcomes became fundamental tools for evaluating performance and identifying opportunities for improvement [10]. Cardiothoracic surgery was particularly well positioned to embrace this approach, having already established a longstanding culture of outcome monitoring, audit, and continuous refinement of surgical techniques [11].

As performance measurement evolved, its purpose gradually expanded beyond internal quality improvement. Outcome data that had traditionally been used within the profession for education and self-assessment increasingly became accessible to external stakeholders, including patients, healthcare administrators, policymakers, and regulatory bodies. Consequently, performance measurement shifted from an internal learning process to a mechanism of public accountability, fundamentally changing both its objectives and its implications for clinical practice.

At the same time, patient engagement came to be regarded not only as an ethical obligation but also as a practical strategy for improving healthcare quality. This perspective was reinforced by market-oriented healthcare reforms, which viewed patients as informed consumers capable of making meaningful choices between providers when provided with reliable comparative information [12]. Public reporting of surgical outcomes therefore emerged at the intersection of quality improvement science, respect for patient autonomy, and healthcare policies promoting transparency, accountability, and patient empowerment.

### 1.3. Aim and Scope of the Review

This narrative review examines the clinical, ethical, and organizational implications of transparency and public reporting in cardiothoracic surgery. It explores both the anticipated benefits of these initiatives—including improvements in quality of care, reinforcement of patient autonomy, and enhancement of public trust—as well as their unintended consequences.

Particular attention is given to the potential adverse effects of public reporting, including risk-averse clinical behaviour, distortion of clinical decision-making, reduced willingness to adopt innovative surgical techniques, and the impact of performance reporting on surgeons’ professional well-being and identity. Building on these considerations, the review proposes an ethically grounded framework for transparency that seeks to balance accountability with fairness, appropriate interpretation of performance data, and the preservation of professional responsibility in contemporary cardiothoracic practice.

Although previous publications have examined public reporting primarily from the perspectives of quality improvement, outcome measurement, or healthcare policy, few have provided an integrated analysis of its clinical, ethical, organizational, and professional implications specifically within cardiothoracic surgery. This review seeks to address that gap by combining current evidence with an ethical framework that emphasizes fairness, shared accountability, responsible interpretation of performance data, and patient-centred transparency.

Because these concepts are closely interconnected, themes such as risk aversion, professional accountability, surgeon well-being, and shared responsibility are revisited throughout the review from complementary clinical, ethical, organizational, and patient-centred perspectives rather than as isolated topics.

**Figure 1:**
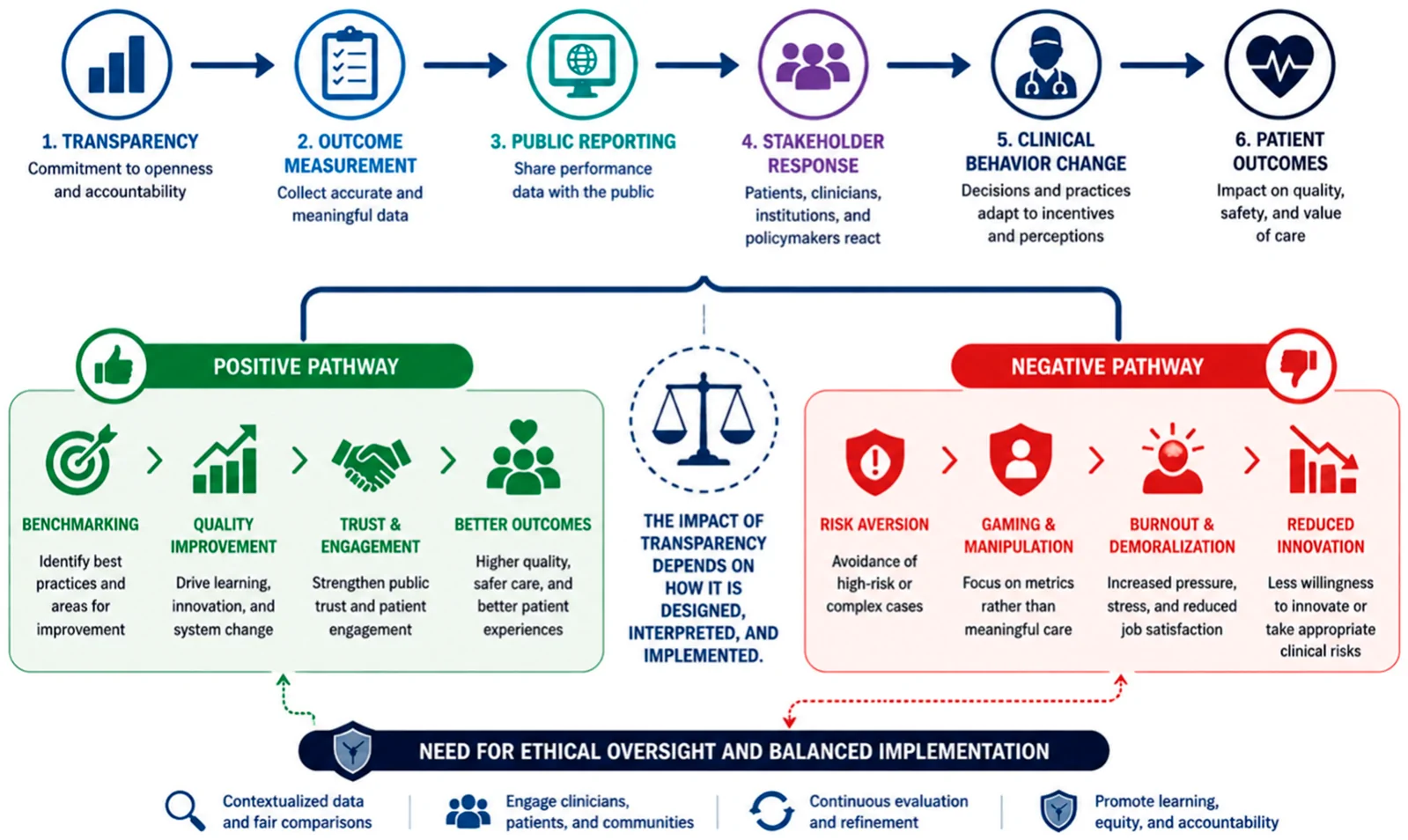
The transparency pathway in cardiothoracic surgery. Conceptual model illustrating how transparency and public reporting may influence healthcare delivery and patient outcomes. The effects of transparency are mediated through outcome measurement, public reporting, stakeholder responses, and subsequent changes in clinical behaviour. Depending on how reporting systems are designed, interpreted, and implemented, transparency may lead to quality improvement and greater public trust, or to unintended consequences such as risk aversion, gaming of performance metrics, professional burnout, and reduced innovation. Ethical oversight and balanced implementation are essential to maximize benefits while minimizing harms.

Importantly, public reporting should not be viewed as an isolated intervention but rather as one component of a broader system influencing patients, clinicians, healthcare institutions, policymakers, and society. Its overall impact depends not only on the publication of performance data but also on how those data are interpreted, communicated, and incorporated into clinical decision-making, institutional governance, and health policy. Figure 1 presents this conceptual framework and illustrates the pathways through which transparency may generate both beneficial outcomes and unintended consequences.

## 2. Materials and Methods

### 2.1. Study Design

This study was conducted as a narrative review to examine the clinical, ethical, and organizational implications of transparency and public reporting in cardiothoracic surgery. A narrative review methodology was selected because the topic encompasses heterogeneous evidence derived from clinical studies, observational research, professional guidelines, policy documents, ethical analyses, and healthcare governance literature. This approach allowed the integration of evidence from multiple disciplines to provide a comprehensive overview of current knowledge and emerging perspectives.

### 2.2. Literature Search Strategy

A literature search was performed using the PubMed and ScienceDirect databases to identify publications relevant to transparency and public reporting in cardiothoracic surgery. The search primarily included articles published between January 2000 and January 2026, although earlier landmark publications were also considered when essential for understanding the historical development of public reporting and transparency initiatives.

Search terms included combinations of the following keywords: “cardiothoracic surgery,” “cardiac surgery,” “thoracic surgery,” “public reporting,” “transparency,” “outcome reporting,” “quality improvement,” “risk adjustment,” “surgical outcomes,” “patient autonomy,” and “accountability.” Reference lists of selected articles were also screened to identify additional relevant publications. The literature search was conducted independently by two authors. Potentially relevant publications were screened by title and abstract, followed by full-text assessment where appropriate. Any differences in study selection were resolved through discussion and consensus.

### 2.3. Study Selection and Data Synthesis

Original research articles, systematic and narrative reviews, clinical practice guidelines, policy documents, consensus statements, and landmark reports were considered for inclusion. Publications were selected based on their scientific relevance to the objectives of this review, with particular emphasis on studies addressing the impact of public reporting on clinical outcomes, quality improvement, risk adjustment, professional practice, ethical decision-making, patient autonomy, transparency, and healthcare governance in cardiothoracic surgery.

The inclusion criteria comprised:

i. Publications addressing transparency, public reporting, outcome measurement, or quality assessment in cardiothoracic surgery or closely related surgical specialties;
ii. Studies examining the clinical, organizational, ethical, or policy implications of public reporting;
iii. Articles published in peer-reviewed journals or official reports from recognized professional societies or governmental organizations;
iv. Publications written in English.

The exclusion criteria included:

i. Articles unrelated to transparency or public reporting in healthcare;
ii. Studies focusing exclusively on technical surgical procedures without relevance to quality assessment or reporting systems;
iii. Conference abstracts, editorials, letters, and commentaries lacking substantial scientific or policy content;
iv. Duplicate publications or studies providing insufficient information to contribute meaningfully to the objectives of the review.

The final selection of references was based on the authors’ assessment of each publication’s scientific relevance, methodological quality, and contribution to the topics under discussion. Because this was a narrative review, no formal systematic review protocol, predefined risk-of-bias assessment, or quantitative meta-analysis was undertaken. Instead, evidence from diverse study designs and publication types was critically synthesized to provide a comprehensive overview of the clinical, ethical, and organizational dimensions of transparency in cardiothoracic surgery.

## 3. Historical Development of Public Reporting in Cardiothoracic Surgery

### 3.1. Early Outcome Tracking and Internal Registries

The origins of outcome tracking in cardiothoracic surgery predate the modern era of public reporting by several decades. Beginning in the 1950s and 1960s, pioneering cardiac surgeons systematically documented their operative outcomes to evaluate emerging surgical techniques, identify opportunities for technical refinement, and improve patient survival [13].

During this early period, outcome data were collected primarily for professional self-assessment and quality improvement rather than for external comparison or public accountability. The intended audience consisted largely of surgeons themselves, fostering a culture in which the analysis of outcomes served as a means of promoting learning and continuous improvement rather than ranking individual performance [14].

Mortality and morbidity conferences gradually became an integral component of surgical practice, providing structured forums for the multidisciplinary review of adverse events and postoperative complications. Within these meetings, complications were viewed primarily as opportunities for collective learning and quality improvement rather than as grounds for blame or professional censure [15]. This approach reflected the longstanding values of the surgical profession, emphasizing peer review, reflective practice, and continuous professional development within a supportive collegial environment.

Internal registries further strengthened these quality improvement efforts by enabling surgeons to monitor their individual learning curves, compare outcomes across different surgical techniques, and identify patients at particularly high operative risk [16]. These registries laid the foundation for the standardized data collection systems and benchmarking initiatives that would later underpin national databases and public reporting programs.

### 3.2. Emergence of National Databases

The establishment of national cardiothoracic surgery databases marked a major milestone in the evolution of surgical quality assessment. These registries transformed the evaluation of clinical outcomes by enabling standardized data collection, institutional benchmarking, and the development of validated risk-adjustment methodologies [11]. Professional societies played a central role in establishing and maintaining these databases, recognizing that systematic outcome monitoring could drive quality improvement across the specialty.

One of the most influential initiatives is the Society of Thoracic Surgeons (STS) National Database. According to the World Health Organization (WHO), the STS National Database, established in 1989, is the world’s largest and most comprehensive cardiac surgery registry [17]. In the United States, participation has expanded from a limited number of surgical programs to more than 1000 surgical groups, representing approximately 95% of all cardiac surgery programs nationwide [18].

The STS database captures detailed information on patient characteristics, operative variables, and clinical outcomes, enabling the development of robust risk prediction models and risk-adjusted comparisons of surgical performance. Similar initiatives have subsequently been established throughout Europe under the leadership of professional societies and national healthcare systems.

Among the most prominent European initiatives are the adult cardiac surgery and congenital heart surgery registries developed by the European Association for Cardio-Thoracic Surgery (EACTS) [19]. In parallel, several countries, including the United Kingdom, Germany, and Sweden, have implemented comprehensive national registries incorporating standardized data collection, risk adjustment, and outcome reporting tailored to their respective healthcare systems [20,21].

The introduction of standardized outcome measures addressed one of the fundamental challenges of comparing surgical performance: distinguishing genuine differences in quality of care from variations arising from inconsistent definitions, differences in case mix, or methodological discrepancies in data collection and analysis [22]. Standardization therefore provided the methodological foundation for fair benchmarking and meaningful comparisons across surgeons, institutions, and healthcare systems.

### 3.3. Transition from Internal Audit to Public Disclosure

As highlighted by Shahian et al. [1], the transition from internal audit to public reporting was driven by evolving healthcare policies, increasing media scrutiny, and growing public expectations regarding transparency and accountability. In the United States, New York State became the first jurisdiction to mandate public reporting of cardiac surgery outcomes in 1989 after administrative data revealed substantial variation in surgical performance [23].

This initiative led to the development of the New York Cardiac Surgery Reporting System (CSRS), which subsequently served as a model for similar reporting programs in other regions. In the United Kingdom, the Bristol Royal Infirmary Inquiry [7] represented another pivotal milestone, highlighting the need for greater transparency in the reporting of surgical outcomes and strengthening public demands for accountability.

Among its key recommendations, the Bristol Inquiry called for the public release of consultant-level outcome data, marking a significant departure from the traditional culture of professional self-regulation [24]. The United Kingdom remains one of the few countries to publish named surgeon outcome data, including individual mortality rates, through the reporting system administered by the Society for Cardiothoracic Surgery in Great Britain and Ireland [21].

The transition to public reporting fundamentally changed both the purpose and the implications of outcome measurement. Data that had previously been collected primarily to support internal quality improvement and professional learning became increasingly relevant to professional reputation, institutional performance, regulatory oversight, and, in some cases, medicolegal accountability [25]. Consequently, the role of transparency expanded well beyond quality assurance, becoming an integral component of contemporary healthcare governance.

The evolution of transparency in cardiothoracic surgery has therefore been gradual, progressing from internal professional audit to national registries, mandatory reporting initiatives, and the comprehensive performance reporting systems used today. The principal milestones in this development are summarized in Table 1.

**Figure 2:**
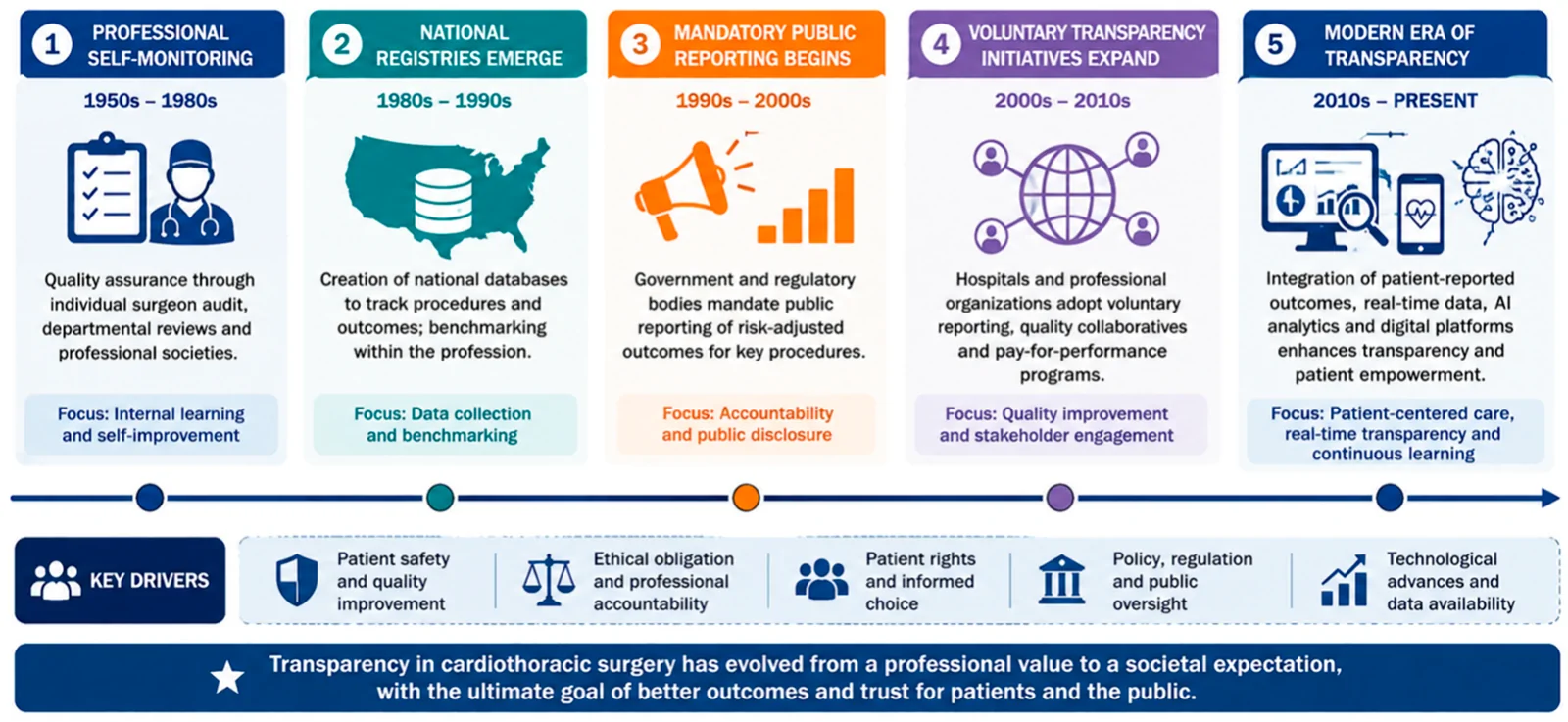
Historical evolution of transparency and public reporting in cardiothoracic surgery. The figure illustrates the transition from professional self-monitoring and internal quality assurance to national registries, mandatory public reporting initiatives, voluntary transparency programs, and contemporary models incorporating patient-reported outcomes, artificial intelligence, and real-time performance monitoring. The key drivers that have shaped this evolution include quality improvement efforts, professional accountability, patient empowerment, regulatory oversight, and technological advances.

The development of transparency in cardiothoracic surgery has been gradual and closely linked to broader changes in healthcare governance. Over the past several decades, the specialty has evolved from a model based primarily on professional self-regulation and internal audit toward systems that increasingly emphasize public accountability, benchmarking, and patient engagement. The major stages of this evolution are illustrated in Figure 2.

## 4. Models of Transparency and Public Reporting

### 4.1. What Is Being Reported?

The outcomes reported through public reporting systems in cardiothoracic surgery vary considerably across countries and healthcare systems. Although reporting frameworks differ, the most commonly reported outcome remains operative mortality, typically defined as death occurring during the index hospitalization or within 30 days of surgery [1].

Mortality is widely regarded as one of the most objective and clinically meaningful outcome measures because it is clearly defined and readily quantifiable. However, mortality alone does not provide a comprehensive assessment of surgical quality. Advances in surgical techniques, perioperative care, and patient management have led to substantial reductions in mortality following many cardiac procedures over recent decades, limiting its ability to discriminate between high- and low-performing providers [17].

To address these limitations, many reporting systems also include morbidity measures that capture major postoperative complications affecting patient recovery and long-term quality of life. Standard morbidity endpoints in cardiac surgery include stroke, renal failure requiring dialysis, prolonged mechanical ventilation, deep sternal wound infection, and reoperation for bleeding or other postoperative complications [14].

Composite outcome measures have emerged as a more comprehensive approach to quality assessment by integrating multiple dimensions of performance into a single risk-adjusted metric [26]. For example, the Society of Thoracic Surgeons (STS) composite performance measures combine mortality, major morbidity, and selected process measures to provide an overall assessment of surgical quality [27].

In addition to outcome-based metrics, some public reporting systems incorporate volume-based indicators, reflecting evidence that higher institutional or surgeon procedural volumes are associated with improved outcomes for selected cardiac surgical procedures [28,29]. Nevertheless, no single measure adequately reflects the complexity of surgical quality. Contemporary reporting systems therefore rely on a combination of complementary outcome, process, and structural indicators to provide a more balanced and comprehensive evaluation of clinical performance.

Cardiothoracic surgery has been at the forefront of specialty-specific quality assessment through the establishment of large clinical registries and structured outcome reporting systems. The Society of Thoracic Surgeons (STS) National Database has become one of the world’s most comprehensive clinical registries, providing robust risk-adjusted outcome data that support benchmarking, quality improvement, and public reporting initiatives [30,31]. Similarly, the European Association for Cardio-Thoracic Surgery (EACTS) Adult Cardiac Surgery Database has contributed substantially to international quality assessment and benchmarking across Europe [32,33]. At the national level, reporting initiatives such as the United Kingdom’s National Adult Cardiac Surgery Audit (NACSA) [34,35] and the German Heart Surgery Report (Deutscher Herzbericht), published annually by the German Society for Thoracic and Cardiovascular Surgery (DGTHG) [36], have further advanced transparency by systematically reporting procedural volumes, outcomes, and quality indicators while promoting continuous quality improvement. In Germany, these efforts are further complemented by several disease- and procedure-specific registries, including the German Registry for Acute Aortic Dissection Type A (GERAADA), the German Aortic Root Repair Registry (GARR), and the German Aortic Valve Registry (GARY), which provide detailed clinical data, facilitate benchmarking, support outcomes research, and contribute to continuous quality improvement within their respective fields [37,38,39]. Together, these specialty-specific registries and national reporting initiatives illustrate how transparent performance reporting can facilitate benchmarking and quality improvement when accompanied by rigorous risk adjustment and careful interpretation of performance metrics.

### 4.2. Who Is Being Reported?

Public reporting systems differ not only in the outcomes they report but also in the level at which performance is evaluated. Reported outcomes may be attributed to individual surgeons, hospitals, healthcare programs, or regional healthcare systems, and the choice of reporting level has important implications for accountability, statistical reliability, and clinical behaviour [1].

The most granular approach involves reporting outcomes at the individual surgeon level, thereby attributing responsibility for surgical performance directly to the operating surgeon. This model has been implemented in several jurisdictions, including New York State, Pennsylvania, and the United Kingdom, where surgeon-specific outcome data are publicly available.

An alternative approach focuses on hospitals or surgical programs rather than individual clinicians. This model recognizes that surgical outcomes reflect not only the technical expertise of the operating surgeon but also the quality of multidisciplinary teamwork, perioperative management, institutional infrastructure, and other organizational factors that collectively influence patient care. Consequently, accountability is assigned to the healthcare institution rather than to individual physicians.

This institutional approach has been adopted by the Society of Thoracic Surgeons (STS) public reporting initiative, in which star ratings are assigned at the participating hospital or program level rather than to individual surgeons [1]. By emphasizing collective performance, this model seeks to encourage system-wide quality improvement while reducing some of the unintended consequences associated with surgeon-specific reporting.

### 4.3. Risk Adjustment and Scoring Systems

Risk adjustment forms the methodological foundation of valid comparisons of surgical outcomes by accounting for differences in patient characteristics that influence outcomes independently of the quality of care [1]. Without appropriate risk adjustment, healthcare providers treating older, sicker, or otherwise more complex patients may appear to perform worse simply because of differences in case mix rather than true differences in clinical performance. Consequently, robust risk-adjustment models are essential for ensuring fair comparisons and reducing incentives for risk selection.

Several validated risk prediction models have been developed for cardiac surgery. Among the most widely used are the European System for Cardiac Operative Risk Evaluation (EuroSCORE) and its successor, EuroSCORE II, both of which estimate the probability of operative mortality based on preoperative patient characteristics [2]. These models enable comparisons between observed and expected mortality, thereby providing a more meaningful assessment of surgical performance across institutions and healthcare systems.

In parallel, the Society of Thoracic Surgeons (STS) has developed a series of procedure-specific risk models derived from the extensive clinical data contained within the STS National Database [27]. These models incorporate numerous patient and procedural variables to support risk-adjusted evaluation of outcomes and have become widely accepted tools for quality assessment and benchmarking.

Despite their sophistication, however, risk-adjustment models are inherently imperfect and cannot completely eliminate bias [40]. Because they can account only for variables that are measured and incorporated into the statistical model, important unmeasured or inadequately captured patient characteristics may still influence outcomes. Such residual confounding can compromise the fairness of provider comparisons and should therefore be considered when interpreting publicly reported performance data.

### 4.4. Presentation Formats

The manner in which performance data are presented to patients, healthcare professionals, policymakers, and the public has a substantial influence on how these data are interpreted and how they affect decision-making [41]. Even when identical outcome data are reported, differences in presentation format can shape perceptions of provider performance and influence clinical, organizational, and patient behaviour.

One of the earliest and most widely recognized presentation formats is the league table, in which providers are ranked from highest to lowest according to a selected performance indicator. Although league tables facilitate straightforward comparisons, they may create an exaggerated perception of performance differences, particularly when statistically insignificant variations are interpreted as meaningful distinctions between providers.

An alternative approach involves the use of star ratings or other categorical grading systems. These methods classify providers into predefined performance categories based on risk-adjusted outcomes and statistical confidence intervals, offering a simplified summary while acknowledging the uncertainty inherent in performance measurement. The Society of Thoracic Surgeons (STS), for example, uses a star rating system to communicate overall institutional performance in a format that is readily understandable to both healthcare professionals and the public [1].

More recently, dashboards and public scorecards have been introduced to provide a more comprehensive representation of healthcare quality. Rather than relying on a single outcome measure, these tools integrate multiple indicators, including mortality, morbidity, process measures, patient safety metrics, and structural characteristics, thereby offering a broader assessment of clinical performance [1].

The effectiveness of any reporting format ultimately depends on the ability of its intended audience to understand and appropriately interpret the information presented. Studies have shown that many members of the public experience difficulties interpreting probabilistic information, confidence intervals, and comparative performance statistics [42]. Consequently, the design of public reporting systems should emphasize clarity, transparency, and appropriate contextualization to minimize misinterpretation and support informed decision-making (Table 2).

## 5. Intended Benefits of Public Reporting

### 5.1. Improving Quality and Outcomes

The primary objective of public reporting is to promote quality improvement by providing performance feedback, facilitating benchmarking, strengthening institutional accountability, and creating reputational incentives for excellence [1]. Although studies evaluating the impact of public reporting in cardiac surgery have produced mixed results, the overall evidence suggests modest but meaningful improvements in clinical outcomes following its implementation.

One of the most influential studies was conducted by Hannan et al. [43], who reported a substantial reduction in risk-adjusted mortality following the introduction of public reporting for coronary artery bypass grafting (CABG) surgery in New York State. Between 1989 and 1992, risk-adjusted mortality declined by 41%, from 4.17% to 2.45%, with improvements observed across providers in all performance terciles. These findings suggest that public reporting may encourage quality improvement not only among lower-performing institutions but across the entire spectrum of surgical providers.

Benchmarking represents one of the principal mechanisms through which public reporting may improve quality. By comparing their outcomes with those of peer institutions, healthcare providers can identify performance gaps, adopt best practices, and implement targeted quality improvement initiatives [44].

The value of benchmarking has been further demonstrated through regional quality collaboratives. Likosky et al. [45] showed that collaborative initiatives, such as the Michigan Society of Thoracic and Cardiovascular Surgeons Quality Collaborative, can successfully use shared performance data to identify opportunities for improvement and promote evidence-based changes in clinical practice. In this context, public reporting serves not only as a mechanism for accountability but also as a catalyst for collective learning and continuous quality improvement.

In addition, reputational incentives may contribute to improved performance by encouraging healthcare organizations to maintain high standards of care and avoid the negative consequences associated with publicly reported poor outcomes [46]. While the magnitude of this effect is difficult to quantify, it represents an important mechanism through which transparency may influence organizational behaviour.

### 5.2. Enhancing Patient Autonomy

A central ethical justification for public reporting is its potential to strengthen patient autonomy by providing individuals with information that can support informed healthcare decisions. Respect for autonomy requires that patients have access to accurate, relevant, and comprehensible information, enabling them to make choices that reflect their personal values, preferences, and treatment goals.

Within this framework, public reporting aims to reduce information asymmetry between healthcare providers and patients by increasing the transparency of surgical outcomes and institutional performance. When presented appropriately, such information can help patients participate more actively in decisions regarding the selection of surgeons, hospitals, and treatment strategies.

This approach is closely aligned with the principles of shared decision-making, a collaborative process in which patients and clinicians combine the best available scientific evidence with the patient’s individual values and preferences to reach informed treatment decisions [47]. In cardiac surgery, decision aids incorporating individualized risk estimates have been shown to improve patients’ understanding of treatment options, facilitate risk communication, and enhance the quality of decision-making [5].

Nevertheless, the ability of public reporting to promote patient autonomy depends not only on the availability of performance data but also on their accessibility, clarity, and appropriate interpretation. Without adequate contextualization, complex outcome statistics may confuse rather than empower patients, thereby limiting the intended benefits of transparency.

### 5.3. Strengthening Public Trust

Transparency has been widely advocated as a means of maintaining and strengthening public trust in healthcare institutions and the medical profession [10]. Trust is closely linked to perceptions of accountability, competence, and integrity. Patients and the wider public are more likely to have confidence in healthcare systems when they believe that clinical performance is systematically monitored, openly evaluated, and subject to appropriate oversight.

Public reporting contributes to this process by making quality assurance and accountability mechanisms more visible. Rather than relying solely on professional self-regulation, transparency demonstrates that healthcare providers and institutions are prepared to evaluate their performance publicly and accept responsibility for the quality of care they deliver. In doing so, public reporting may reinforce confidence that poor performance will be identified, investigated, and addressed through appropriate quality improvement measures.

Nevertheless, transparency alone is not sufficient to generate public trust. Its effectiveness depends on the accuracy of the reported data, the fairness of performance evaluation, and the clarity with which information is communicated. When these conditions are met, public reporting can strengthen confidence in healthcare institutions and support the broader legitimacy of healthcare governance.

## 6. Unintended Clinical Consequences

### 6.1. Risk Aversion and Case Selection

The most consistently documented unintended consequence of public reporting is risk aversion, defined as the tendency of healthcare providers to avoid or decline treatment for patients perceived to be at high operative risk because unfavorable outcomes may negatively influence publicly reported performance measures [40]. This phenomenon, often referred to as risk selection or “cherry-picking,” raises important concerns regarding equitable access to care, particularly for patients with the greatest clinical need.

Survey-based studies have repeatedly demonstrated that public reporting can influence clinical decision-making among cardiac surgeons [48]. In a landmark survey, 66% of cardiac surgeons in New York reported that they would be less likely to operate on a high-risk patient requiring coronary artery bypass grafting (CABG)—a procedure subject to public reporting—than on a patient with an equivalent operative risk undergoing repair of an acute aortic dissection, for which outcomes were not publicly reported. These findings suggest that reporting systems may unintentionally create incentives for selective case acceptance based on whether procedural outcomes are publicly monitored.

Evidence of risk-averse behaviour has also been observed at the population level. Several studies have reported lower rates of coronary revascularization among high-risk patients in states with mandatory public reporting compared with states without such reporting programs [49]. These findings have raised concerns that transparency initiatives, despite their intended benefits, may inadvertently discourage intervention in patients who stand to benefit most from surgical treatment.

Recognizing these unintended consequences, modifications to public reporting policies have been introduced in some jurisdictions. For example, the exclusion of patients presenting with myocardial infarction complicated by cardiogenic shock from publicly reported mortality statistics has been associated with a reduction in risk-averse behaviour. Nevertheless, concerns remain that some high-risk patient groups may continue to experience undertreatment because of the perceived impact of adverse outcomes on publicly reported performance measures [50].

### 6.2. Impact on Innovation and Surgical Courage

Public reporting may also have unintended consequences for surgical innovation by discouraging the adoption of novel techniques or the treatment of patients requiring complex or unconventional procedures. Surgical innovation inevitably involves a period of technical refinement during which outcomes may be less predictable than those achieved with well-established procedures. Consequently, surgeons working in environments where performance is publicly reported may be reluctant to undertake innovative interventions if early results could adversely affect their publicly available outcome data.

This concern extends beyond technological innovation to the broader concept of surgical courage. Surgical courage has been defined as the willingness to offer operative treatment to patients who may derive substantial benefit from surgery despite presenting exceptional technical complexity or a high baseline operative risk [51]. Such decisions require careful clinical judgment, technical expertise, and a balanced assessment of the potential risks and benefits for each individual patient.

Public reporting may unintentionally discourage this aspect of surgical practice by creating incentives to avoid cases associated with uncertain or potentially unfavorable outcomes. Although transparency aims to improve accountability and quality of care, excessive emphasis on publicly reported performance metrics may shift decision-making toward risk minimization rather than individualized patient benefit. Over time, this could limit both surgical innovation and access to advanced therapies for patients with the most complex clinical conditions.

### 6.3. Distortion of Clinical Judgment

Public reporting has the potential to influence clinical judgment by creating incentives to prioritize performance metrics over individualized patient care [52]. When publicly reported outcomes become an important measure of professional performance, clinicians may consciously or unconsciously focus on optimizing measurable indicators while giving less attention to important aspects of care that are not captured by existing reporting systems.

One manifestation of this phenomenon is the adoption of defensive clinical decision-making, whereby treatment recommendations are influenced not only by the patient’s best interests but also by concerns about the potential impact of adverse outcomes on publicly reported performance [53]. Under these circumstances, surgeons may become more conservative in recommending operative treatment for patients with borderline indications or elevated operative risk, even when surgery may offer the greatest potential clinical benefit.

Such behavioural changes illustrate that the effects of public reporting extend beyond quality measurement and accountability alone. While transparency can promote benchmarking, patient engagement, and public confidence, it may also influence clinical decision-making, professional behaviour, and equitable access to surgical care in unintended ways. Achieving the benefits of transparency therefore requires reporting systems that support high-quality, patient-centred decision-making without creating incentives that discourage appropriate treatment of complex or high-risk patients. The principal benefits and potential drawbacks of public reporting in cardiothoracic surgery are summarized in Table 3.

**Figure 3:**
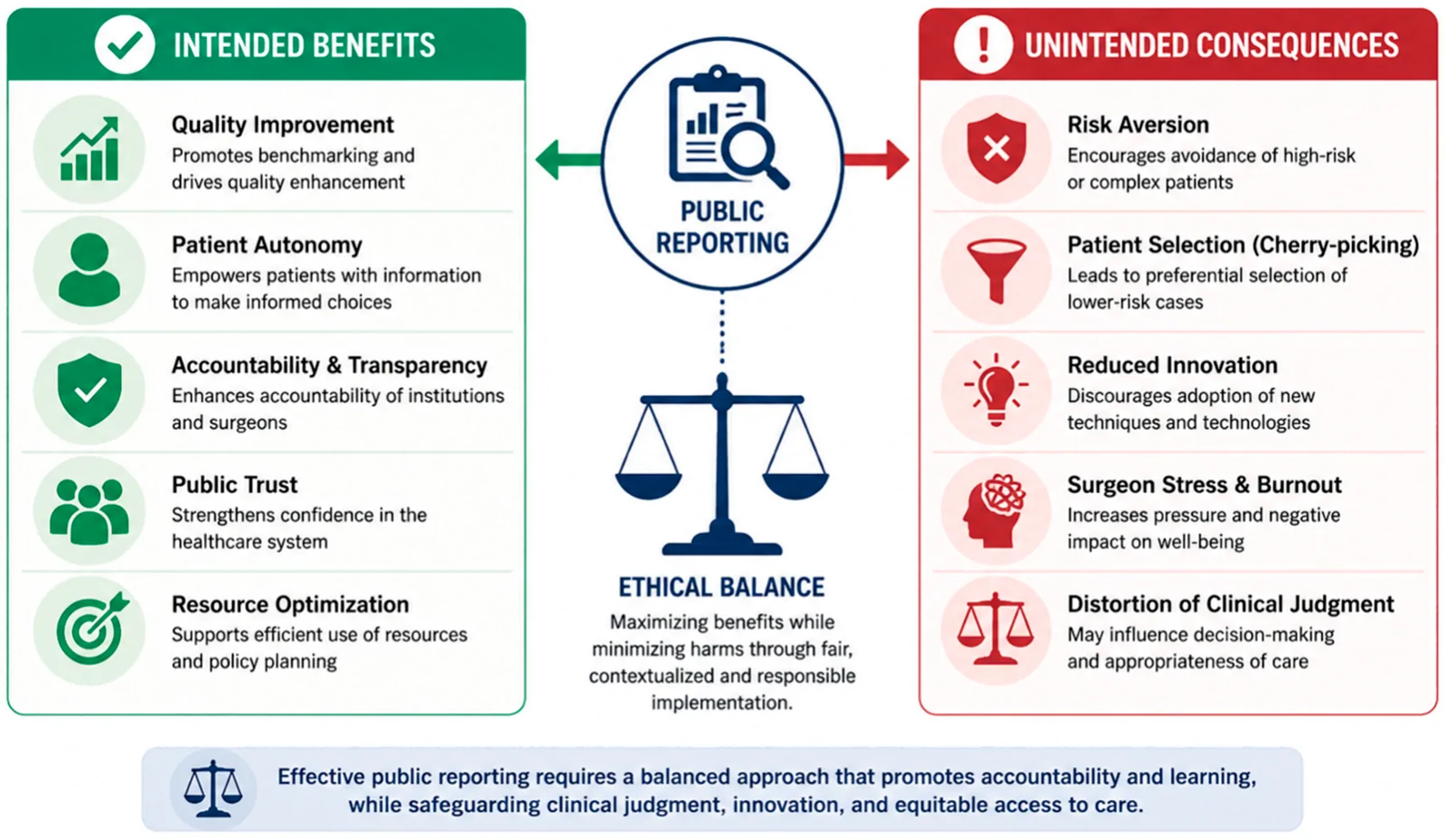
Intended benefits and unintended consequences of public reporting in cardiothoracic surgery. Public reporting may improve accountability, quality improvement, patient autonomy, and public trust. However, it may also contribute to risk-averse behaviour, selective case acceptance, reduced innovation, increased professional stress, and distortions in clinical decision-making. Achieving an appropriate balance between these competing effects remains one of the central ethical challenges of contemporary transparency initiatives.

The effects of public reporting extend beyond simple measurements of quality and accountability. While transparency may promote benchmarking, patient empowerment, and public trust, it can also influence clinical behaviour in unintended ways. The relationship between these competing effects is illustrated in Figure 3, which highlights the need to balance the benefits of public reporting against its potential adverse consequences.

## 7. Ethical Analysis of Public Reporting

### 7.1. Transparency vs. Justice

One of the central ethical challenges of public reporting is balancing the principles of transparency and justice [40]. Transparency seeks to promote accountability by making healthcare outcomes publicly available, whereas justice requires that healthcare professionals and institutions be evaluated fairly, taking into account the circumstances in which care is delivered. Achieving both objectives simultaneously remains one of the greatest challenges in the design of public reporting systems.

The ethical tension arises because no risk-adjustment model can completely account for differences in patient complexity or clinical context. When outcome comparisons fail to fully reflect variations in case mix, publicly reported performance may unfairly disadvantage providers caring for the sickest and most medically complex patients while favoring those whose patient populations are associated with lower baseline risk. Under these circumstances, transparency may inadvertently undermine rather than promote distributive justice.

This concern is particularly relevant for surgeons practicing in safety-net hospitals, tertiary referral centres, and academic medical institutions, where patients frequently present with advanced disease, multiple comorbidities, and substantially higher operative risk than those treated in less complex healthcare settings [49]. Such providers may therefore appear to have inferior outcomes despite delivering care of equal—or even superior—quality.

These challenges are further amplified by socioeconomic and referral-related disparities. Patients from disadvantaged backgrounds often present later in the course of disease, have a greater burden of comorbid conditions, and may experience limited access to social and community resources that support postoperative recovery. Many of these factors influence surgical outcomes independently of the quality of care but are only partially captured by contemporary risk-adjustment models [54]. Consequently, ethical implementation of public reporting requires not only transparency but also continuous refinement of risk adjustment and careful interpretation of outcome data within their broader clinical and social context.

### 7.2. Responsibility and Moral Burden

Public reporting has also altered perceptions of professional responsibility by shifting accountability for surgical outcomes increasingly from the healthcare team to the individual surgeon [55]. Although cardiothoracic surgery is inherently multidisciplinary, the publication of surgeon-specific outcome data may create the impression that adverse outcomes are attributable primarily to a single clinician rather than reflecting the collective contributions of the surgical team, perioperative care, institutional resources, and patient-related factors. This shift raises important ethical questions regarding the fair attribution of responsibility.

The personalization of outcome reporting can impose a substantial moral and psychological burden on surgeons. Adverse clinical outcomes are an unavoidable reality of high-risk surgical practice, and surgeons frequently experience feelings of guilt, self-doubt, and emotional distress following complications or patient deaths. When these outcomes are publicly reported and directly linked to an individual surgeon, the psychological impact may be intensified by concerns regarding professional reputation, public perception, and career progression [56].

Persistent exposure to this level of scrutiny may have important consequences for professional well-being. Over time, the cumulative emotional burden associated with adverse outcomes and public accountability may contribute to burnout, moral injury, emotional exhaustion, and, in some cases, premature departure from clinical practice. These consequences affect not only individual surgeons but also the sustainability of the cardiothoracic workforce and the long-term resilience of healthcare systems.

From an ethical perspective, accountability should remain proportionate and acknowledge the collaborative nature of modern surgical care. While individual surgeons must accept responsibility for their clinical decisions and technical performance, adverse outcomes should be interpreted within the broader context of multidisciplinary teamwork, institutional processes, and patient-related risk factors. Public reporting systems should therefore seek to promote accountability without imposing an excessive moral burden on individual clinicians or discouraging the care of patients with complex or high-risk conditions.

### 7.3. The Illusion of Objectivity

Public reporting systems present surgical performance primarily through quantitative indicators, such as mortality rates, complication rates, and composite performance scores. Although these measures provide valuable information, their numerical precision may create an illusion of objectivity, suggesting a level of certainty and comparability that exceeds what the underlying data can realistically support [57].

When patients, policymakers, or healthcare administrators interpret these metrics as definitive indicators of quality, they may overlook the methodological assumptions, statistical uncertainty, and contextual factors inherent in performance measurement. Differences in case mix, institutional resources, referral patterns, and other unmeasured variables may substantially influence outcomes despite sophisticated risk-adjustment models. Consequently, numerical performance indicators should be interpreted as estimates rather than absolute measures of clinical quality.

This limitation reflects the broader challenge of statistical abstraction, whereby complex clinical realities are reduced to numerical summaries. Although such simplification is necessary for benchmarking and public reporting, it inevitably omits important contextual information surrounding individual patients, clinical decision-making, and organizational factors that influence surgical outcomes. As a result, quantitative measures cannot fully capture the complexity of clinical practice or the quality of care delivered in individual cases.

From an ethical perspective, transparency should therefore encompass not only the publication of performance data but also clear communication of their limitations and appropriate interpretation. Public reporting is most ethically defensible when it promotes informed understanding rather than an unwarranted perception of precision or certainty.

### 7.4. Transparency as Moral Virtue or Moral Hazard

Transparency occupies a complex position within healthcare ethics and may be viewed from two contrasting ethical perspectives. On the one hand, transparency is widely regarded as a moral virtue because it reflects honesty, integrity, accountability, and respect for the legitimate interests of patients, healthcare professionals, and society in accessing meaningful information about healthcare quality [58]. From this perspective, openly reporting clinical outcomes demonstrates professional responsibility and reinforces public confidence in healthcare institutions.

Conversely, transparency may also function as a moral hazard if the incentives it creates undermine the very goals it is intended to achieve [59]. When publicly reported performance measures encourage risk-averse behaviour, distort clinical judgment, discourage innovation, or limit access to care for high-risk patients, transparency may inadvertently compromise patient welfare despite its well-intentioned objectives. In such circumstances, the ethical value of transparency cannot be judged solely by its intention to promote accountability but must also be evaluated according to its practical consequences for patients, clinicians, and healthcare systems.

These contrasting perspectives suggest that transparency should not be regarded as an absolute ethical good. Rather, its moral legitimacy depends on how it is designed, implemented, and interpreted within clinical practice. Public reporting is ethically justified only when it promotes accountability while preserving fairness, supporting patient-centred decision-making, encouraging innovation, and protecting equitable access to high-quality surgical care.

## 8. Effects on Surgeons and Surgical Culture

### 8.1. Professional Identity and Autonomy

Public reporting has fundamentally influenced the professional identity of cardiothoracic surgeons by shifting the focus from traditional professional autonomy toward greater external accountability [55]. Historically, surgeons were regarded as highly skilled professionals whose practice was guided primarily by technical expertise, clinical judgment, and peer evaluation. Contemporary public reporting systems, however, increasingly assess surgical performance through standardized outcome metrics that are publicly accessible and subject to external scrutiny.

This transformation reflects a broader change in the culture of surgical practice. Throughout much of the twentieth century, professional excellence was evaluated largely through peer recognition, clinical experience, and self-reflection within the surgical community. Although these elements remain essential, public reporting has introduced quantitative performance indicators as an additional means of assessing quality. While such measures improve transparency and accountability, they may not fully capture important aspects of surgical practice, including clinical judgment, technical complexity, individualized decision-making, and the management of exceptionally challenging cases.

As a result, some surgeons perceive a gradual shift in professional autonomy, with increasing emphasis placed on measurable outcomes rather than the broader competencies that characterize high-quality surgical care. From an ethical perspective, this raises important questions regarding how professional excellence should be defined and assessed. A balanced approach should recognize the value of objective performance measurement while acknowledging that not all dimensions of surgical expertise can be adequately quantified.

### 8.2. Burnout, Moral Injury, and Fear of Failure

The psychological consequences of public reporting have received increasing attention alongside growing recognition of burnout and moral injury among physicians [60,61]. Although public reporting is neither the sole nor the primary cause of psychological distress among cardiothoracic surgeons, it may amplify existing pressures within a profession characterized by high clinical responsibility, emotionally demanding decision-making, and an inherent risk of adverse outcomes.

The public disclosure of surgical performance introduces an additional layer of scrutiny that may intensify anxiety regarding complications, mortality, and professional reputation. For many surgeons, adverse outcomes are already associated with feelings of guilt, self-doubt, and emotional distress. When these outcomes are publicly reported and linked to individual performance, concerns about reputation, career progression, and public perception may further increase psychological stress [56].

Public reporting may also contribute to moral injury, which arises when clinicians feel unable to act in accordance with their professional and ethical obligations because of external pressures. In this context, surgeons may experience moral conflict if concerns about publicly reported outcomes influence decisions to decline operative treatment for high-risk patients who might nevertheless benefit from surgery. Such situations create tension between the ethical duty to provide the best possible care for each individual patient and the desire to avoid adverse outcomes that could negatively affect publicly reported performance.

Over time, persistent exposure to these pressures may contribute to chronic stress, emotional exhaustion, burnout, and reduced professional satisfaction. These effects have implications not only for surgeon well-being but also for workforce sustainability, recruitment, and retention within cardiothoracic surgery. Consequently, efforts to promote transparency should be accompanied by measures that support surgeons’ psychological well-being while preserving a culture of learning, professional resilience, and patient-centred care.

### 8.3. Training and Mentorship Implications

Public reporting may also have important implications for surgical education and the training of future cardiothoracic surgeons [62]. The development of surgical competence depends on progressive participation in increasingly complex procedures under appropriate supervision. Inevitably, this learning process is associated with a learning curve during which operative efficiency and clinical outcomes may differ from those achieved by highly experienced surgeons.

Concerns have been raised that public reporting may unintentionally reduce trainee involvement in complex surgical procedures because supervising surgeons seek to minimize the potential impact of adverse outcomes on publicly reported performance metrics [63]. This phenomenon, often referred to as risk-transfer avoidance, describes the reluctance to entrust technically demanding cases to trainees when doing so is perceived to increase the likelihood of unfavorable publicly reported outcomes.

Restricting trainee participation in high-complexity cases may have important long-term consequences for surgical education. Reduced operative exposure can delay the acquisition of technical skills, limit clinical decision-making experience, and ultimately affect the preparedness of future cardiothoracic surgeons to manage complex patients independently. From an ethical perspective, balancing patient safety, transparency, and the educational mission of teaching hospitals represents a continuing challenge.

To address these concerns, public reporting systems should acknowledge the unique role of academic centres in surgical training and encourage educational environments in which supervised trainee participation can occur without creating inappropriate disincentives. Supporting high-quality surgical education while maintaining accountability is essential for ensuring both current patient safety and the future sustainability of the specialty.

## 9. Patient Perspectives and Misinterpretation of Data

### 9.1. Statistical Literacy and Public Understanding

The effectiveness of public reporting ultimately depends not only on the availability of performance data but also on the ability of patients and the general public to understand and appropriately interpret the information provided. Public reporting can only support informed decision-making if its intended audience is able to access, comprehend, and meaningfully use the reported data. However, numerous studies have shown that many individuals experience difficulty interpreting probabilistic information, risk estimates, confidence intervals, and comparative performance statistics, raising concerns about the effectiveness of current reporting formats [64].

Health literacy and statistical literacy therefore play a central role in determining whether public reporting achieves its intended objectives. Complex numerical data may be misinterpreted, leading patients to overestimate small differences in performance or to draw conclusions that are not supported by the underlying evidence. Without appropriate explanation and contextualization, performance metrics may inadvertently increase confusion rather than facilitate informed healthcare choices.

The manner in which information is presented further influences how it is interpreted. This phenomenon, commonly referred to as the framing effect, has been shown to substantially affect perceptions of risk, benefit, and treatment quality [65]. For example, identical clinical outcomes may be perceived differently depending on whether they are presented as survival rates or mortality rates, despite conveying equivalent statistical information. Consequently, the design of public reporting systems should prioritize clear communication, intuitive presentation formats, and appropriate contextual information to support accurate interpretation and informed patient decision-making.

### 9.2. Trust and the Doctor-Patient Relationship

Public reporting has the potential to influence the doctor–patient relationship by shaping patients’ perceptions of competence, accountability, and trustworthiness [66]. Trust is a fundamental component of effective clinical care, particularly in cardiothoracic surgery, where patients must often make decisions involving substantial operative risks and place considerable confidence in their surgical team.

When public reporting demonstrates that healthcare providers and institutions are committed to transparency, quality assessment, and continuous improvement, it can strengthen patient confidence and reinforce trust in both individual clinicians and the healthcare system. Transparent communication of outcomes may reassure patients that performance is subject to independent evaluation and that mechanisms exist to promote accountability and maintain high standards of care.

However, transparency does not automatically foster trust. If performance data are presented without adequate explanation, appropriate risk adjustment, or sufficient clinical context, patients may misinterpret the information or lose confidence in providers whose outcomes appear unfavorable despite caring for more complex patient populations. In such circumstances, transparency may inadvertently undermine rather than strengthen the therapeutic relationship.

Ultimately, the impact of public reporting on trust depends not only on the availability of performance data but also on how those data are communicated, contextualized, and discussed within the clinical encounter. Transparent, honest, and patient-centred communication remains essential for preserving the trust that underpins shared decision-making and high-quality surgical care.

### 9.3. Shared Decision-Making in the Era of Scorecards

Effective shared decision-making in cardiothoracic surgery requires the integration of three essential components: the best available clinical evidence, the individual patient’s values and preferences, and the specific clinical circumstances of each case [5]. Publicly reported performance data can contribute to this process by providing additional information regarding institutional or procedural outcomes. However, such data should complement rather than replace individualized clinical judgment and patient-centred discussion.

Decision aids incorporating personalized risk estimates have been shown to improve patients’ understanding of treatment options, facilitate risk communication, and enhance the quality of shared decision-making in cardiac surgery [47]. By presenting individualized estimates of operative risk alongside evidence-based treatment options, these tools help patients make decisions that are better aligned with their goals and expectations.

Nevertheless, publicly reported outcome data often require careful interpretation within the broader clinical context. Differences in patient characteristics, procedural complexity, and risk adjustment may not be readily apparent to patients reviewing scorecards or performance reports. Consequently, clinicians play a pivotal role in helping patients understand the meaning, limitations, and appropriate use of publicly reported information.

In the era of public reporting, shared decision-making should therefore be viewed as an interpretive process rather than a simple comparison of performance metrics. Publicly available outcome data are most valuable when integrated with individualized risk assessment, professional clinical expertise, and open communication between patients and clinicians. This approach enables transparency to support—not replace—patient-centred decision-making.

## 10. Transparency in the Age of Artificial Intelligence

Although the application of artificial intelligence to public reporting in cardiothoracic surgery remains at an early stage, recent advances in machine learning, predictive analytics, and clinical decision support suggest that AI is likely to play an increasingly important role in outcome prediction, risk adjustment, and quality assessment.

### 10.1. AI-Augmented Public Reporting

Advances in artificial intelligence (AI) and machine learning are transforming healthcare quality assessment and creating new opportunities—as well as new challenges—for public reporting in cardiothoracic surgery [67]. AI-based analytical tools have the potential to enhance existing reporting systems by improving risk prediction, identifying patterns in large clinical datasets, and facilitating more sophisticated assessments of surgical performance.

Unlike conventional regression-based risk models, machine learning algorithms can incorporate substantially larger numbers of variables and identify complex, non-linear relationships that may improve predictive accuracy and more effectively account for patient heterogeneity [68]. These capabilities may enable more precise risk adjustment, thereby improving the fairness and reliability of comparisons between surgeons and institutions.

AI also offers the possibility of shifting public reporting from periodic, static reports toward continuously updated performance dashboards. Automated data collection and real-time analytics could provide clinicians, healthcare organizations, and patients with more timely information regarding outcomes, quality indicators, and emerging performance trends. Such systems may facilitate earlier identification of quality concerns and support continuous quality improvement rather than retrospective performance assessment.

However, the integration of AI into public reporting also raises important ethical and practical considerations. Machine learning models are frequently criticized for their limited interpretability, creating concerns about transparency, reproducibility, and accountability when performance assessments are generated by complex algorithms. In addition, algorithmic bias resulting from incomplete or unrepresentative training data may inadvertently reinforce existing disparities or produce unfair evaluations of surgeons or institutions caring for higher-risk patient populations. Nevertheless, robust prospective validation, external validation across diverse healthcare systems, and careful regulatory oversight will be essential before AI-assisted public reporting can be implemented routinely in clinical practice. Consequently, AI should be viewed as a tool to support—rather than replace—clinical expertise, transparent governance, and human oversight in performance evaluation.

### 10.2. New Risks of Algorithmic Transparency

The integration of AI into public reporting introduces new risks that require careful consideration. One important concern is the use of algorithm-derived performance measures that lack transparency or interpretability, often referred to as “black-box” metrics [69]. When clinicians and patients cannot understand how these metrics are generated, confidence in the fairness and reliability of performance evaluations may be undermined.

Another major concern is algorithmic bias. Machine learning models are trained on historical data, which may contain existing biases related to patient populations, clinical practice, or healthcare access. If left unaddressed, these biases may be perpetuated or even amplified, leading to unfair comparisons between surgeons or institutions and potentially reinforcing existing healthcare disparities [68]. At present, empirical evidence evaluating the real-world impact of AI-assisted public reporting remains limited, and many proposed applications have yet to undergo rigorous clinical evaluation.

### 10.3. Diffusion of Responsibility

The integration of artificial intelligence into public reporting raises important questions regarding accountability that existing frameworks are not well equipped to address. When algorithm-generated performance ratings are used to inform high-stakes decisions, it can be difficult to determine responsibility for errors or unintended consequences. Current models of professional and institutional accountability were developed for human decision-making and may require adaptation to address the challenges posed by AI-assisted performance evaluation.

## 11. Toward Ethical Transparency: A Balanced Framework

The preceding sections demonstrate that transparency is neither inherently beneficial nor inherently harmful. Rather, its impact depends largely on how public reporting systems are designed, implemented, and interpreted. These considerations provide the foundation for a balanced framework of ethical transparency in cardiothoracic surgery.

### 11.1. Principles for Responsible Public Reporting

The ethical framework proposed in this review aims to establish principles that balance accountability with fairness, learning with evaluation, and patient interests with professional sustainability. Accurate interpretation of performance data requires appropriate contextualization, including case-mix information, the limitations of risk adjustment, and the inherent uncertainty of outcome measures.

Continuous investment in the development and validation of risk-adjustment models is essential to ensure fair comparisons among providers treating different patient populations. Furthermore, transparency systems should be designed primarily to promote quality improvement rather than to identify and penalize poor performers, thereby fostering a culture of learning rather than punishment [69].

### 11.2. Human-in-the-Loop Transparency

The principle of human-in-the-loop transparency emphasizes that transparency systems should not be viewed as self-interpreting sources of quantitative performance data. Human interpretation should remain an essential component of the evaluation process [70]. Clinicians play a key role in providing the context necessary for patients to understand performance data. In addition, mechanisms for ethical oversight should be established to identify and address unintended consequences of transparency systems.

### 11.3. Reframing Transparency as Shared Accountability

Reframing transparency as shared accountability represents not only a shift from ranking-based reporting to meaningful accountability, but also an important ethical reorientation. Shared accountability recognizes that outcomes in cardiothoracic surgery reflect the combined contributions of individual clinicians, multidisciplinary teams, healthcare institutions, and the broader health system, rather than the performance of a single provider alone.

**Figure 4:**
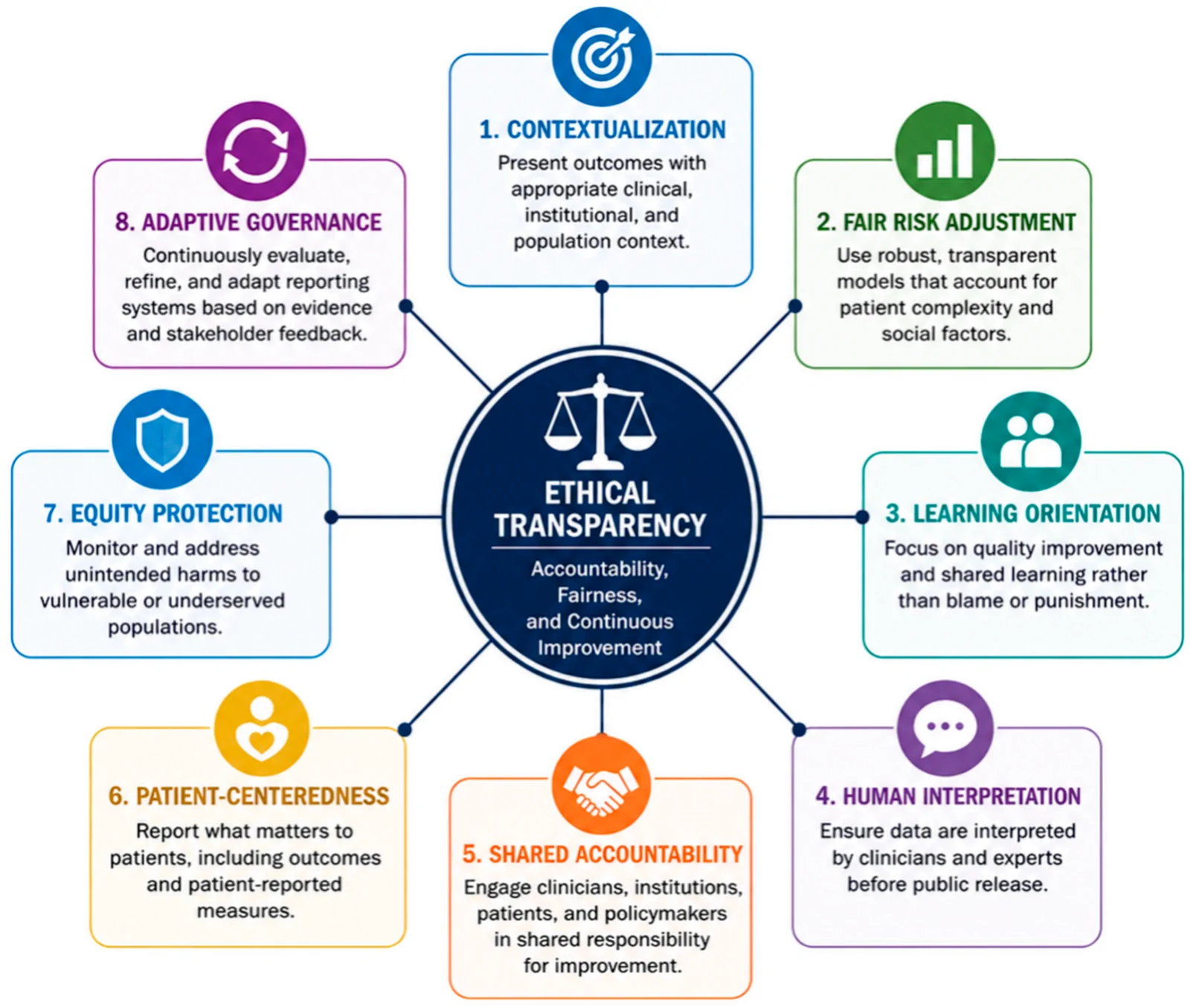
Proposed framework for ethical transparency in cardiothoracic surgery. Ethical transparency should extend beyond the simple publication of outcome data. A balanced reporting system should incorporate contextualization, fair risk adjustment, learning orientation, human interpretation, shared accountability, patient-centredness, equity protection, and adaptive governance. Together, these principles aim to promote accountability while preserving fairness, professional integrity, and continuous quality improvement.

By shifting the focus from exposure to improvement, transparency evolves from a mechanism of potential punishment into a tool for quality enhancement. The preceding discussion highlights the need for a more balanced approach to transparency in cardiothoracic surgery. Public reporting should promote accountability and quality improvement without creating unfair incentives, compromising access to care, or undermining professional judgment. The proposed framework for ethical transparency, illustrated in Figure 4, is based on a set of complementary principles intended to support responsible and patient-centred reporting practices.

The principles proposed in this review aim to support a model of transparency that promotes accountability while preserving fairness, professional integrity, patient-centred care, and opportunities for continuous improvement. The key elements of this framework are summarized in Table 4.

## 12. Future Directions

### 12.1. Smarter Metrics

Developing metrics that better reflect the complexity of surgical quality and the diversity of patient goals will further advance transparency in cardiothoracic surgery. Incorporating additional measures of case complexity beyond traditional risk variables may enable fairer comparisons among providers treating different patient populations.

Patient-reported outcome measures (PROMs) offer an opportunity to capture outcomes that matter most to patients, including functional status, symptom relief, and quality of life [71,72,73,74].

Future developments should continue to build upon established specialty-specific registries, including those maintained by the STS and EACTS, which provide high-quality clinical data capable of supporting increasingly sophisticated risk-adjustment models, patient-centred outcome measures, and responsible public reporting [30,31,32,33]. Continued collaboration between national and international registries will be essential for improving data quality, benchmarking, and the comparability of reported outcomes across healthcare systems.

### 12.2. Patient-Centered Reporting

Redesigning public reporting with patient-centeredness as its guiding principle could improve its usefulness for the decisions patients actually face. The primary purpose of public reporting should be to support informed decision-making rather than simply facilitate comparisons between providers. Moreover, the outcomes that matter most to patients may differ from those traditionally reported publicly.

### 12.3. Integration with Ethical Governance

To ensure sustainable transparency, public reporting should be integrated into broader ethical governance frameworks that promote ongoing attention to unintended consequences, fairness, and alignment with healthcare values. Institutions play a key role in bringing stakeholders together, establishing standards, and monitoring transparency systems. In addition, safeguards should be implemented to protect providers from the most severe unintended negative effects of public reporting.

### 12.4. Remaining Challenges and Areas of Ongoing Debate

Despite the growing body of evidence supporting greater transparency in healthcare, several important areas remain the subject of ongoing debate. The overall impact of public reporting may vary considerably depending on healthcare systems, reporting methodologies, risk-adjustment models, and institutional cultures. Likewise, concerns persist regarding unintended consequences, including risk-averse clinical behaviour, potential misinterpretation of performance metrics, administrative burden, and the challenges of accurately capturing surgical quality through quantitative indicators alone. These ongoing controversies highlight that transparency should not be viewed as a universally applicable solution but rather as an evolving process requiring continuous evaluation, refinement, and ethical oversight to ensure that it improves patient care while minimizing unintended consequences.

## 13. Limitations

Several limitations of this review should be acknowledged. First, this is a narrative review and, therefore, does not follow the methodology of a systematic review or meta-analysis. Although the literature search was designed to identify the most relevant publications on transparency and public reporting in cardiothoracic surgery, study selection inevitably involved a degree of author judgement.

Second, much of the available evidence originates from specific healthcare systems, particularly the United States and the United Kingdom, where public reporting initiatives have been established for many years. Consequently, some observations may not be fully generalizable to healthcare systems with different organizational structures, funding mechanisms, or regulatory environments.

Third, several topics discussed in this review, including professional identity, moral burden, patient trust, and ethical accountability, are difficult to quantify and are often examined through qualitative research, policy analyses, or theoretical frameworks. As a result, some conclusions are necessarily interpretative rather than based solely on measurable clinical outcomes.

Finally, the landscape of healthcare transparency continues to evolve. Emerging technologies, including artificial intelligence-based performance assessment and real-time outcome reporting, may substantially change the opportunities and challenges associated with public reporting in the coming years. Future research is needed to evaluate the long-term impact of these developments on patients, healthcare professionals, and healthcare systems.

## 14. Conclusions

Transparency through public reporting has become a permanent feature of cardiothoracic surgery, driven by legitimate demands for accountability, quality improvement, and patient empowerment. This review demonstrates that public reporting can improve patient outcomes, strengthen patient autonomy, and enhance public accountability. At the same time, the available evidence highlights important unintended consequences that require ongoing attention, including risk-averse decision-making that may limit access to surgery for high-risk patients, distortions of clinical judgment, reduced opportunities for surgical innovation, and psychological burdens that may contribute to burnout and moral injury. By integrating clinical evidence, ethical analysis, and emerging developments such as artificial intelligence, this review provides a comprehensive framework for implementing transparency in a manner that supports both patient care and professional practice. In cardiothoracic surgery, transparency is necessary but not sufficient to ensure high-quality care. The value of public reporting depends not only on what is reported, but also on how performance data are generated, interpreted, and used. Ethical transparency requires balancing several legitimate goals: accountability to patients and the public, fairness for providers caring for complex patient populations, support for innovation, and preservation of the trust that underpins the therapeutic relationship. Taken together, these recurring themes underline the multifaceted nature of transparency, illustrating that its effects extend beyond outcome reporting to encompass professional behaviour, ethical decision-making, organizational culture, and patient care. Ultimately, transparency should guide better care rather than merely generate more data.

## Figures and Tables

**Table 1:** Historical Evolution of Transparency and Public Reporting in Cardiothoracic Surgery: Key Milestones, Drivers, and Reporting Objectives.

Era	Period	Key Developments	Primary Drivers	Reporting Focus
Professional Self-Monitoring	1950s–1980s	Mortality and morbidity conferences; internal registries; peer review	Professional culture; learning from complications	Internal quality improvement; educational purposes
Emergence of National Databases	1980s–1990s	STS National Database (1989); EACTS registries; standardized definitions	Professional societies; benchmarking needs	Institutional comparison; risk model development
Mandatory Public Reporting	1989–2000s	New York CSRS (1989); Pennsylvania reporting; Bristol Inquiry (UK)	Policy pressure; media attention; patient advocacy	Public accountability; consumer information
Voluntary Professional Reporting	2000s–2010s	STS public reporting initiative; SCTS named surgeon data (UK)	Professional leadership; quality improvement	Star ratings; composite measures; transparency
Contemporary Era	2010s–present	Integration with value-based care; AI-augmented analytics; PROMs	Healthcare reform; technology advancement	Patient-centered outcomes; real-time monitoring

**Table 2:** Principal Metrics and Risk-Adjustment Methods Used in Public Reporting of Cardiothoracic Surgical Outcomes.

Category	Specific Metrics	Advantages	Limitations
Mortality Measures	Operative mortality; 30-day mortality; in-hospital mortality	Objective; clinically meaningful; readily understood	Low event rates limit discrimination; may not capture all quality dimensions
Morbidity Measures	Stroke; renal failure; prolonged ventilation; deep sternal wound infection; reoperation	Captures non-fatal complications; broader quality assessment	Definitions may vary; ascertainment challenges
Composite Measures	STS CABG composite; STS valve composites; multiprocedural composites	Improved statistical reliability; comprehensive assessment	Complex methodology; may obscure individual domain performance
Volume Indicators	Annual procedural volume; surgeon volume; hospital volume	Simple to measure; evidence of volume-outcome relationship	Causal mechanisms debated; may disadvantage smaller programs
Process Measures	IMA use in CABG; perioperative medication adherence	Directly actionable; evidence-based	May not correlate with outcomes; gaming potential
Risk Adjustment Models	EuroSCORE II; STS risk models; institutional models	Enable fair comparisons; account for case-mix	Cannot adjust for unmeasured confounders; calibration drift

**Table 3:** Intended Benefits and Potential Unintended Consequences of Public Reporting in Cardiothoracic Surgery.

Domain	Intended Benefits	Unintended Consequences
Quality of Care	Benchmarking drives improvement; identification of outliers; motivation for institutional investment	Gaming of data; focus on measured rather than unmeasured quality; statistical noise misinterpreted as signal
Patient Autonomy	Informed decision-making; empowerment; respect for persons	Statistical illiteracy limits utility; false reassurance or undue alarm; undermines trust when unexplained
Clinical Practice	Evidence-based care; standardization; reduced unwarranted variation	Risk aversion; cherry-picking; conservative treatment recommendations; delayed or denied care for complex patients
Surgical Innovation	Accountability for experimental approaches; safety monitoring	Reduced willingness to attempt novel procedures; suppression of surgical courage; slower adoption of advances
Professional Culture	Transparency as professional virtue; accountability to society	Moral injury; burnout; fear of failure; transition from craftsman to metric subject
Training	Outcome awareness for trainees; quality-focused education	Reduced trainee autonomy; risk transfer avoidance; compromised educational opportunities
Equity	Identification of disparities; attention to underserved populations	Penalization of safety-net providers; reduced access for high-risk patients; exacerbation of disparities

**Table 4:** Proposed Ethical Framework for Responsible Transparency and Public Reporting in Cardiothoracic Surgery.

Principle	Description	Implementation Strategies
Contextualization	Performance data should be presented with sufficient information about case-mix, limitations, and uncertainty	Include confidence intervals; provide narrative context; acknowledge risk adjustment limitations; present trends over time
Fair Risk Adjustment	Comparisons should account for patient characteristics that influence outcomes independently of care quality	Continuous model refinement; validation across subgroups; attention to unmeasured confounders; socioeconomic variable inclusion
Learning Orientation	Transparency systems should prioritize quality improvement over identification and punishment of poor performers	Confidential feedback mechanisms; quality collaborative participation; focus on improvement trajectories; support for struggling programs
Human Interpretation	Quantitative data should be complemented by clinical interpretation and ethical oversight	Clinician involvement in explaining data; ethics review of reporting policies; stakeholder input in system design
Shared Accountability	Responsibility for outcomes should be distributed appropriately across all contributors to care	Team-level reporting; institutional rather than individual attribution; recognition of system factors; interdisciplinary quality improvement
Patient-Centeredness	Reported outcomes should reflect what matters most to patients, not merely what is convenient to measure	Incorporation of PROMs; patient involvement in metric selection; decision support rather than ranking focus; meaningful outcome definitions
Equity Protection	Transparency systems should not disadvantage providers serving vulnerable populations or exacerbate health disparities	Monitoring for differential effects; adjustment for social determinants; protection for safety-net providers; access preservation
Adaptive Governance	Transparency policies should be monitored for unintended consequences and modified as evidence accumulates	Ongoing evaluation; stakeholder feedback mechanisms; flexibility in implementation; research on reporting effects

## Data Availability

No new data were created or analyzed in this study. Data sharing is not applicable to this article.
